# Synonymous but Not Equal: A Special Section and Virtual Issue on Phenotypic Effects of Synonymous Mutations

**DOI:** 10.1093/gbe/evab186

**Published:** 2021-09-01

**Authors:** Casey McGrath

It has been 50 years since Francis Crick, Leslie Barnett, Sydney Brenner, and Richard Watts-Tobin first deduced the nature of the genetic code, revealing how a gene’s nucleotide sequence is related to the encoded protein’s amino acid sequence. Essential to cracking the code was the premise that the genetic code is degenerate: as there are 64 possible codons (i.e., nucleotide triplets) and only 20 standard amino acids, some amino acids must be specified by multiple codons. One consequence of this degeneracy is that some mutations alter DNA sequences without changing the underlying protein sequence. Although it was long believed that these “synonymous” mutations were effectively neutral, half a century later, genome-wide analyses have begun to reveal both the evolutionary forces that shape synonymous mutations as well as their potential phenotypic effects. In a new Special Section titled “Phenotypic Effects of Synonymous Mutations,” guest editors Stéphanie Bedhomme and Ignacio Bravo have gathered four articles that compile some of the mechanisms through which synonymous mutations can result in phenotypic effects. In addition, an accompanying virtual issue highlights an additional eight articles on this topic published in “Genome Biology and Evolution” over the past three years, providing an even broader look at recent insights in this growing field.

Providing a compelling overview of this topic, the Special Section includes a review article by Bailey et al. (2021) that highlights what microbial experimental evolution has revealed about the contribution of synonymous mutations to adaptation. Although selection for rapid and highly efficient translation is among the most commonly postulated drivers of codon usage bias (the preferential use of certain codons over others to encode the same amino acid), Bailey and colleagues discuss how synonymous mutations can “impact fitness through a range of mechanisms including the creation of illicit RNA polymerase binding sites impacting transcription and changes to mRNA folding stability that modulate translation.” Indeed, another review in the Special Section by Callens et al. (2021) (coauthored by guest editor Bedhomme) discusses how codon usage may be shaped by selective pressures to avoid or enrich for specific sequence motifs such as: transcription and translation initiation and termination signals, mRNA maturation signals, and antiviral immune system targets.

The remaining two articles in the Special Section focus on the unique forces shaping synonymous mutations in viruses, which rely on their host’s translational machinery. In Pintó and Bosch (2021), the authors discuss how local codon usage bias can control the rate of translation, thereby altering protein folding and function. Mordstein et al. (2021) analyze over 1,500 viral genomes and reveal that codon usage patterns in viruses reflect a combination of selective and mutational pressures related to the viral life cycle and RNA or DNA genome nature, including the need for efficient transcription, protein export, and immune evasion.

Building on this theme, two papers in the virtual issue also examine the effects of synonymous mutations in viruses. In their study, Van Leuven et al. (2021) take a reverse engineering approach, systematically deoptimizing all nonoverlapping genes of the bacteriophage ΦX174 by replacing existing codons with those rarely used in the virus’ host *Escherichia coli*. Intriguingly, the resulting reductions in viral fitness are neither linear nor additive within a given gene, and are further not consistent between genes, indicating the need for a better understanding of how selection acts on synonymous codons across genes and genomes. In another paper coauthored by Pintó and Bosch, D’Andrea et al. (2019) elucidate fitness landscapes of the hepatitis A virus to reveal the importance of the codon composition of viral capsid genes in regulating translation and determining the virus’s robustness.

Since tRNAs are responsible for interacting with and “decoding” codons, there is thought to be a clear interplay between codon usage and tRNA abundance or copy number in a genome, especially for fast-growing unicellular organisms. However, investigating codon usage patterns and tRNA gene copy numbers in Gammaproteobacteria, Mahajan and Agashe (2018) conclude that selection on translational speed alone cannot fully explain the variation in codon usage bias observed in this lineage. Even more intriguing, Ottenburghs et al. (2021) reveal that bird tRNA genes are reduced in number and complexity compared with those of other vertebrates, suggesting unique selective pressures on synonymous mutations in this lineage. 

At an even higher phenotypic level, since the seminal works by Toshimichi Ikemura, who investigated the relationship between codon usage and tRNA abundance in the early 1980s (Ikemura 1985), there has been a presumed relationship between codon usage and gene expression. Indeed, Yannai et al. (2018) show that selection on codon usage is evident in both highly and lowly expressed genes in *E. coli*, including the 10% of genes expressed at the very lowest levels. Moreover, they demonstrate that a single synonymous mutation within a lowly expressed but essential gene can have a considerable effect on fitness. In a comparison of over 2,000 *Drosophila melanogaster* genes, each expressed in a single tissue, Payne and Alvarez-Ponce (2019) reveal that genes exhibit tissue-specific codon usage patterns, which they attribute to differences in tRNA abundances across tissues, reflecting different translational selection regimes.

The final two papers in the virtual issue examine the evolutionary pressures and phenotypic effects of synonymous mutations in more specific scenarios. Based on genomic data from primates, Mier and Andrade-Navarro (2018) show that differential evolutionary forces act on stretches of DNA encoding 1–3 consecutive glutamine residues compared with those encoding 4–8 glutamines in a row. The existence of such localized forces acting on specific codons suggests there may be many more of these small-scale patterns yet to be elucidated. Finally, in a paper coauthored by the Special Section guest editors Bedhomme and Bravo, the authors investigate the effects of synonymous differences on horizontally transferred genes (Bedhomme et al. 2019): the horizontal transfer to *E. coli* of three synonymous versions of an antibiotic resistance gene produced contrasting levels of resistance. Experimental evolution of these lines led to substantial genomic and proteomic changes, dependent on the version used, but all leading to a high level of resistance. This indicates the potential of synonymous differences to shape posthorizontal transfer evolution.

Together, the articles in this Special Section and virtual issue provide a strong foundation for further studies on the phenotypic effects of synonymous mutations, not only establishing a current framework for such analyses, but also posing outstanding questions and new avenues for exploration on this topic.
